# The Impact of Health and Social Care Integration on Children and Young People’s Outcomes: What Can Be Determined from Scotland’s Administrative Data?

**DOI:** 10.5334/ijic.9145

**Published:** 2025-11-26

**Authors:** Joanna Soraghan, Alexander McTier, Micky Anderson, Carol Ann Anderson, Emma Young, Adrian Bowman, Heather Ottaway

**Affiliations:** 1 CELCIS (Centre for Excellence for Children’s Care and Protection), University of Strathclyde, UK; 2 School of Mathematics and Statistics, University of Glasgow, UK

**Keywords:** integration, outcomes, children’s services, multilevel modelling, integrated care, Scotland

## Abstract

**Introduction:**

The integration of services is often driven by the belief that integration will lead to better outcomes for service users. However, there is a paucity of robust evidence exploring the relationship between integration and outcomes. This study sought to determine whether the integration of health and social care services via Health and Social Care Partnerships has led to a measurable change in outcomes for Scotland’s children and young people.

**Methods:**

Multilevel models were applied to routinely collected administrative data to determine whether different approaches to structural integration were related to changes in a range of outcomes for children and young people. The modelling approach accounted for confounding factors such as economic conditions and the COVID-19 pandemic.

**Results:**

The analysis found no consistent evidence of an association between the structural integration of services and changes in outcomes for children and young people. However, external factors such as deprivation and the COVID-19 pandemic were found to be linked to changes in outcomes across various areas of children’s lives.

**Conclusions:**

The findings highlight the complexity in attributing changes in outcomes to a specific intervention or reform, particularly in the presence of wider socio-economic factors. Understanding the influence of systems-level change may not be fully possible using routinely collected data alone, and any methods used to assess impact should be underpinned by an underlying theory of change.

## Introduction

Structural integration, where previously separate organisations merge to create a new joint entity that governs, manages and resources staff and services, is described as the most developed form of multi-agency working [1,2]. It is also viewed as the optimal approach to integrated working, portrayed as entirely benign and leading to improvements in system efficiency, effectiveness and service user outcomes [1,2,3,4,5].

The positivist belief in the benefits of structural integration has shaped how many countries have responded to increasing levels and complexity of demand for children’s services. Influenced by developments in adult health and social care, there has been a trend towards establishing more integrated and outcomes-focused children’s services systems [2,6,7]. The policy assumption is that more integrated structures leads to more timely, joined up and seamless support from different services, which in turn helps to meet the needs of children and improve their wellbeing and outcomes. Examples of this policy assumption being enacted include the establishment of Children’s Trust Pathfinders in England [5,8], Wellbeing Service Counties in Finland [9], Health and Social Care Trusts in Northern Ireland [10], Child and Family Units in Norway [11], and the national children and family agency (Tusla) in the Republic of Ireland [12].

The evidence base underpinning the policy assumption is, however, limited, with theories of change that articulate how structural change leads to improved children’s experiences and outcomes widely lacking [1,2,4,7,13,14,15]. This article provides an important contribution by presenting the findings from a statistical analysis developed to understand the effects of health and social care integration in Scotland on a range of children’s outcomes. In the context that the ‘purpose of health and social care integration is to transform people’s experience of care and the outcomes they experience’ [16], the article introduces Health and Social Care Partnerships (HSCPs) in Scotland, describes the statistical model developed to assess the effects of integration, and considers the research’s implications in terms of our collective understanding of the relationship between integration and outcomes.

## Background

Health, social care and education are devolved matters in Scotland, and the closer integration of these services has been central to Scotland’s approach to improving children’s outcomes [3,17,18]. In 2006, *Getting It Right For Every Child* [19] was introduced as the national framework to encourage and enable services and practitioners to work together to meet the needs of individual children. In 2011, the Commission on the Future Delivery of Public Services stated that ‘Public service providers must be required to work much more closely in partnership, to “integrate service provision”’ [20, p. VI], warning also that reforms should be driven by how best to achieve improved outcomes, ‘otherwise, we risk bearing the significant costs of structural change, without reaping any real rewards’ [20, p. X]. In response to the Commission’s report, two key pieces of legislation were passed. The first was The Public Bodies (Joint Working) (Scotland) Act 2014 which mandated the integration of adult health and social care services into 32 local HSCPs, with the integration of children’s health, social work and care services into these structures optional. The second was The Children and Young People (Scotland) Act 2014 which required closer collaboration between local authorities and health boards, including jointly producing Integrated Children’s Services Plans.

Allowing time for these new integrated structures and arrangements to form, no major policy and legislative developments concerning structural integration followed until the Independent Review of Adult Social Care in Scotland was announced by the Scottish Government in 2020. The resulting report recommended that a National Care Service for adult social care be created, quoting insufficient progress and ‘unacceptable’ regional variation under the HSCP model as key reasons for change [21, p. 43]. A Scottish Government consultation on the development of a National Care Service was launched in August 2021, including a proposal that children’s social work, social care and community health services be integrated within the National Care Service. The proposed inclusion of children’s social work and care services was somewhat unforeseen because Scottish policy and legislation to that point had centred on more collaborative working arrangements rather than structural integration.

The consultation results found broad support for the National Care Service in relation to adult social care but were inconclusive in relation to the integration of children’s social work and care services [22]. Many respondents asked for further detail on the arrangements for and benefits of including children’s social work and care services. The Scottish Government acknowledged this [23] and stated that further evidence was required. As part of the evidence base, the Scottish Government commissioned the Children’s Services Reform Research (CSRR) study [24]. The statistical analysis presented in this paper formed one of the study’s four research strands and was designed to address the evidence gap surrounding the impact of integration on outcomes for children. The other strands of the research comprised a rapid evidence review [25], international case studies of transformational reform programmes [26] and a survey, focus groups and interviews with Scotland’s children’s services workforce about their experiences of integrated working [27].

The need for greater evidence on the relationship between structural integration of children’s services and improved children’s outcomes is not specific to Scotland [2,3,14]. The only study identified in the international literature that investigated the statistical relationship between structural integration and children’s outcomes was the evaluation undertaken of England’s Children’s Trust Pathfinders [5]. While acknowledging that their analysis could only assess change in children’s indicators up to one year after the Children’s Trust Pathfinders were established, O’Brien and colleagues [5] found no evidence of improvements in children’s outcomes that were directly attributable to the new structures. The Audit Commission came to a similar conclusion, stating ‘there is little evidence that children’s trusts, as required by the government, have improved outcomes for children and young people or delivered better value for money, over and above locally agreed cooperation’ [28, p. 4]. The lack of evidence of impact led to the Conservative-Liberal Democrat coalition government dismantling Children’s Trusts in 2010 [8].

HSCPs are a different structure to England’s Children’s Trusts, as HSCPs are primarily focused on the local integration of adult health and social care services, with the integration of children’s health services and/or children’s social work and care services optional. Nevertheless, the methodology used by O’Brien and colleagues [5] offered important learning for this research and highlighted the challenges involved in investigating the statistical relationship between structural integration and children’s outcomes, particularly where a theory of change does not exist. Theories of change are a critical resource in the design, implementation, monitoring and evaluation of change programmes as they articulate the aims, target beneficiaries, activities, outputs and outcomes of the change, and the connections between these, as well as setting out the measures for tracking and evidencing progress and impact [29].

One challenge is the lack of an agreed set of children’s outcomes indicators. Children’s outcomes may exist in policy, for example in Scotland children’s outcomes are set as children who are safe, healthy, achieving, nurtured, active, respected, responsible and included – often abbreviated to the acronym SHANARRI [19] – but an accompanying set of statistical indicators to measure performance against each of these outcomes was yet to be developed. The Accounts Commission and Auditor General [17] and the Scottish Government [30] agreed nine indicators to monitor the performance of HSCPs in relation to *adults* but none were specified in relation to children.

Another challenge highlighted by O’Brien [5] is the complexity in interpreting change within children’s outcome indicators. For a number of indicators, the direction of travel that would constitute an improvement is not immediately clear. For example, a decrease in the number of children in out of home care may widely be seen as an improvement, but it could also mean that more children are living in unsafe home environments and that their needs are not being met. A theory of change would enable the direction of each indicator to be considered within its wider context.

Indicators should also be sensitive to the organisational and practice changes brought about by integration [5]. A theory of change would again assist by helping to focus on indicators that relate to the effects of structural integration. Where sensitive indicators are identified, there is then the challenge of how to attribute improvements to the effects of structural integration [3,4,6,13] as opposed to any wider contextual changes.

## Methods

The aim of this research was to assess whether there is a relationship between the structural integration of adult and children’s health and social care services across Scotland’s 32 local authority areas, and changes in children’s outcomes within those areas. This was possible because the optional integration of children’s services had led to variations in HSCP structures [18]. This was helpful from a methodological perspective as, consistent with other comparative analyses [1,5], performance and impact could be compared between different structural forms. The research required the following steps:

1. Categorisation of the varying approaches taken to the integration of children’s health and social care services within HSCPs
2. Selection of outcome indicators for children across a variety of domains
3. Statistical modelling to determine if change in the indicators was associated with the different approaches taken to integration, while adjusting for other factors that may concurrently influence children’s outcomes

### Categorisation of the varying approaches to integration

**Figure 1:**
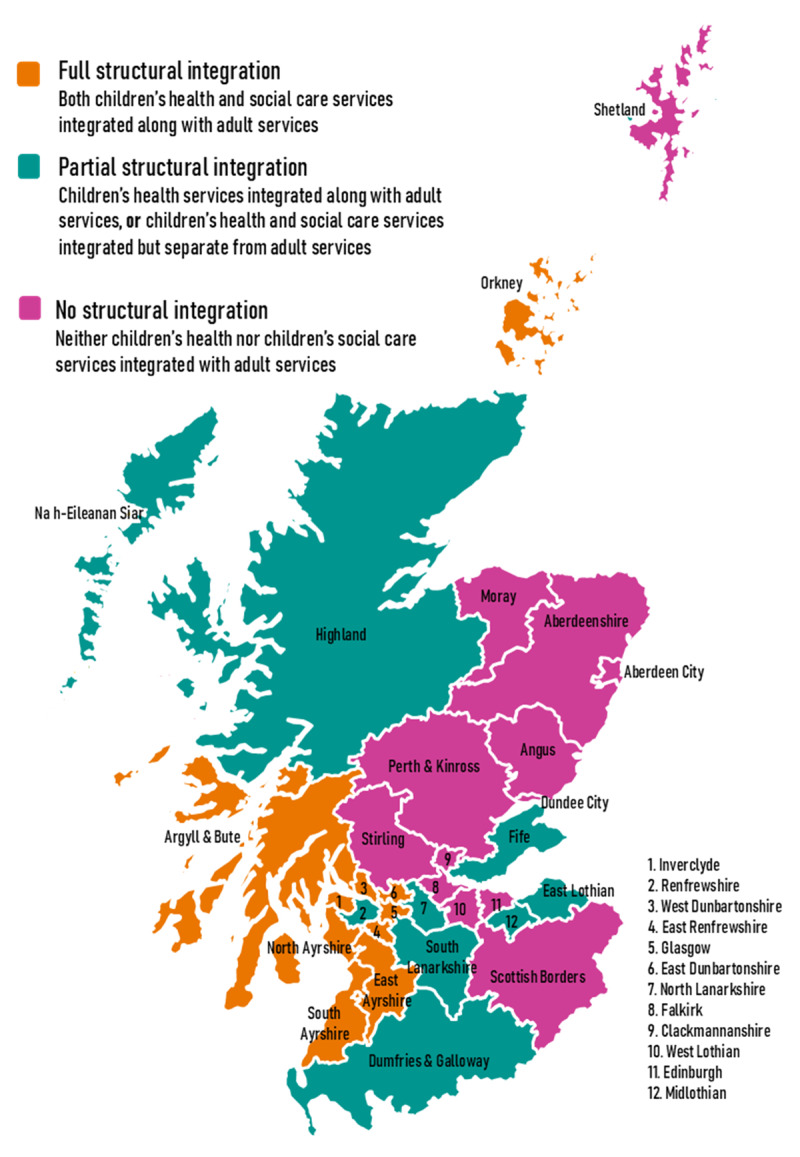
Level of structural integration of children’s services within Scotland’s HSCPs during our period of study to 2021.

To understand the variations in HSCP structures, information published by Health and Social Care Scotland [31] was collated and verified. Three distinct approaches to the integration of children’s services were identified (see Table 1), with the geographical distribution of these approaches shown in Figure 1.

**Table 1:** Defined categories for the integration of children’s services into Scotland’s HSCPs.


DEFINED CATEGORY OF INTEGRATION	DESCRIPTION OF CATEGORY	NUMBER OF LOCAL AUTHORITY AREAS WITH THIS ARRANGEMENT

Full structural integration	Both children’s health and social care services integrated into the HSCP alongside adult’s health and social care services	10

Partial structural integration	Only children’s health services integrated into the HSCP alongside adult’s health and social care services	9

No structural integration	Neither children’s health nor social care services integrated into the HSCP alongside adult’s health and social care services	13


### Selection of outcome indicators

To select a set of children’s outcome indicators from the available administrative data, the research team initially undertook a review of the children’s data held and published by national data producers, including the Scottish Government, Public Health Scotland and the Scottish Children’s Reporter Administration. The review led to 39 potential indicators being identified, covering areas such as health, education, youth justice, housing and child protection. These indicators were then assessed for inclusion against the following criteria:

– The indicator relates to an outcome that could be expected to be impacted by integration
– The data is available for each of the 32 local authority areas
– The data is available over an extended time series, ideally pre-dating 2016 when most HSCP integration in Scotland began, but as a minimum from 2016.
– The data is of sufficient quality in terms of completeness and consistency of recording across local authority areas and time series.

After assessment against these criteria, 25 indicators were selected for inclusion in the analysis (see Table 2). As can be seen in the table, there were several indicators available that related to the Safe, Achieving, Healthy and Nurtured outcomes of the Scottish Government’s SHANARRI framework, however no indicators were identified for the Active, Responsible, Respected or Included outcomes.

**Table 2:** Association of the level of structural integration (and other contextual factors) with changes in each indicator.


INDICATOR	INDICATOR APPLIED TO GIRFEC SHANARRI OUTCOMES	MEASURE	YEARS AVAILABLE AT THE TIME OF ANALYSIS	*P*-VALUE FOR THE ASSOCIATION BETWEEN THE LEVEL OF INTEGRATION AND CHANGES IN THE INDICATOR	CONTEXTUAL FACTORS FOUND TO BE ASSOCIATED WITH CHANGES IN THE INDICATOR		

					COVID–19	DEPRIVATION	POPULATION DENSITY

**CHILD PROTECTION**							

Child protection registrations (including pre-birth)	Safe	Rate per 10,000	9 (2013-2021)	.939	^↓c^	^↑^	^↓^

Child protection de-registrations	Safe	Rate per 10,000	9 (2013-2021)	.454	–	^↑^	–

Case conference to child protection registration conversion rate (0-15 years)	Safe	%	9 (2013-2021)	**.010**	^↓c^	–	^↓^

Children’s Hearings arranged for children for non-offence grounds	Safe	Rate per 10,000	9 (2013-2021)	.257	^↓c^	^↑^	^↓^

**YOUTH JUSTICE**							

Children referred to Children’s Reporter on offence grounds	Safe	Rate per 10,000	11 (2011-2021)	.279	^↓c^	–	–

Children and young people aged 12 to 20 proceeded against	Safe	Rate per 10,000	6 (2016-2021)	.063^b^	^↓c^	^↑^	–

**‘LOOKED AFTER’ CHILDREN**							

Children starting to become looked after	Safe	Rate per 10,000	11 (2011-2021)	.461	^↓c^	^↑^	–

Children starting to be looked after at home as proportion of all children becoming looked after	Safe	%	11 (2011-2021)	.852	^↓c^	–	–

Children ceasing to be looked after (0-15 years)	Safe	Rate per 10,000	12 (2010-2021)	.257	–	^↑^	–

Children aged 0-15 leaving care to return home	Safe	%	12 (2010-2021)	.257	–	^↑^	^↓^

Children with 3+ placements in last 12 months	Safe	%	12 (2010-2021)	**.010**	^↓c^	–	–

Looked after school leavers with 1+ qualifications as SCQF level 4	Achieving	%	11 (2011-2021)	.883	–	^↓^	–

Looked after school leavers with a positive follow-up destination	Achieving	%	11 (2011-2021)	.852	–	^↓^	^↑^

School attendance for looked after children	Achieving	%	4^a^ (2015-2021)	.301^b^	–	–	–

**EDUCATION AND EMPLOYABILITY**							

Unauthorised absence rates of primary school pupils	Achieving	% of half days	6^a^ (2011-2021)	.461	^↓c^	^↑^	–

Unauthorised absence rates of secondary school pupils	Achieving	% of half days	6^a^ (2011-2021)	.131	–	^↑^	–

16-19 year olds not in education, training or employment	Achieving	%	6 (2016-2021)	.257^b^	^↑c^	^↑^	^↑^

**HEALTH**							

Teenage pregnancy rate, under-18	Healthy	Rate per 10,000	10 (2010-2019)	.317	–	^↑^	^↑^

Primary 1 children (4-6 years) overweight or obese	Healthy	%	9 (2011-2019)	**.015**	–	–	–

Children (0-17 years) registered with an NHS dentist	Healthy	%	12 (2010-2021)	.126	^↓c^	–	–

27-30 month old children reviewed by health visitors	Healthy	%	8 (2014-2021)	.900^b^	^↓c^	–	^↑^

27-30 month old children with a developmental concern	Healthy	%	8 (2014-2021)	.588^b^	^↑c^	^↑^	–

**HOUSING**							

Children associated with applications assessed as homeless or threatened with homelessness	Nurtured	Rate per 10,000	11 (2011-2021)	.126	^↓c^	^–^	–

Children in temporary accommodation	Nurtured	Rate per 10,000	12 (2010-2021)	.852	–	^↓^	^↑^

**WORKFORCE**							

Whole-time equivalent rates for social workers in fieldwork services for children	N/A	Rate per 100,000	12 (2010-2021)	.979	–	^↑^	–


^a^ Data published every two years. ^b^ Data for the indicator was only available after integration. The stated *p*-value represents the evidence of an association between the level of integration and *performance* on the indicator in the post-integration period (as opposed to *changes* in the indicator post-integration). ↑ ^c^ Higher values seen in the indicator during COVID-19, ↓ ^c^ Lower values seen in the indicator during COVID-19, ↑An increase in the contextual factor was associated with an increase in the value of the indicator, ↓An increase in the contextual factor was associated with a decrease in the value of the indicator, – No significant association found between the contextual factor and the indicator.

### The association between the level of integration and changes in children’s outcome indicators

To identify any potential relationship between service integration and changes in children’s outcome indicators, a series of multilevel models were fitted to the data. This modelling approach was selected to account for the longitudinal nature of the data, arising from repeated annual measurements from each of Scotland’s 32 local authorities. An overview of multilevel models and their use is provided by Brown [32].

**Figure 2:**
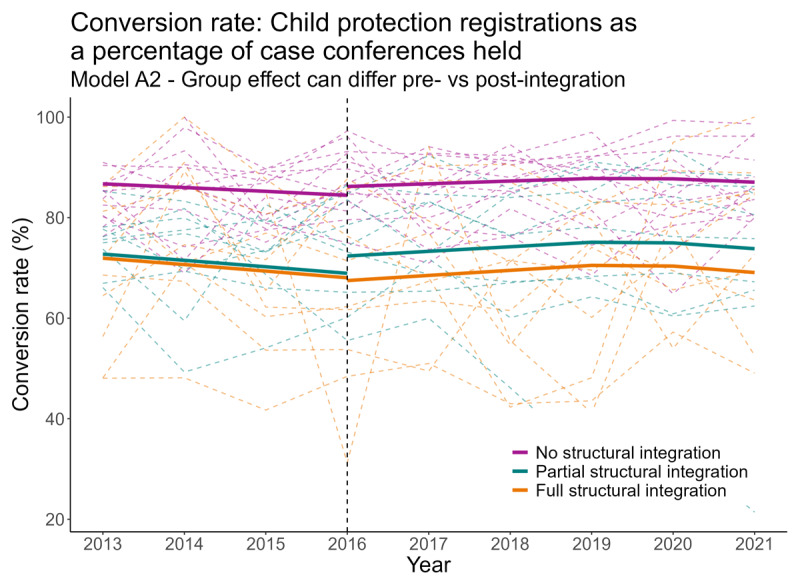
The conversion rate of case conferences to child protection registrations (for 0-15 year olds).

The models were designed to determine the trend for the local authority areas in each of the three defined categories (Full structural integration, Partial structural integration and No structural integration) both prior to and after HSCP integration took place (see example in Figure 2). This approach accounted for the fact that the local authorities within each of the three integration categories may be starting from a different baseline for any given indicator. This could be either due to random fluctuations, or something more systematic such as greater capabilities to integrate services in higher-performing local authority areas or, conversely, reticence to make structural changes where services were already performing well.

It was important that the multilevel model accounted for additional factors that may be driving either an improvement or deterioration in the outcome indicators. As such, information was collected on a variety of potential confounding variables (or ‘contextual factors’) which were then adjusted for within the statistical modelling. The contextual factors were selected as they are widely understood to have an impact on many of the indicators [33,34,35,36,37], and were:

1. Deprivation: the levels of deprivation within each local authority area as measured by the Scottish Index of Multiple Deprivation [38];
2. Population density: calculated using Mid-Year Population Estimates published by National Records of Scotland [39] and the area in km^2^ of local authorities as provided by the Office for National Statistics [40];
3. Co-terminous administrative boundaries: whether the health board covered one or multiple local authority areas;
4. Impact of the COVID-19 pandemic: an indicator variable specifying whether specific data points had been recorded during the COVID-19 pandemic.

The inclusion of these contextual factors increased confidence that any changes in the outcome indicators that had resulted from other causes would not incorrectly be attributed to the effects of integration, while also providing additional insight into which factors had been most influential to children’s outcome indicators over the study period.

For each of the 25 outcome indicators, the modelling process led to a *p*-value representing the evidence for an association between the level of integration of children’s services and changes in the indicator after integration. Due to the large number of indicators being assessed, the *p*-values were adjusted for multiple comparisons using the Benjamini-Hochberg method [41] to minimise the risk of a Type I error occurring. After adjustment, *p*-values lower than the standard statistical threshold of *p* < .05 were taken as evidence of a significant association between structural integration and changes in the values of that particular indicator. Where a significant association was found, the changes detected in the indicator were explored further through the calculation of estimated marginal means for each category of integration both prior to and after integration.

Full details on the modelling process, including the specification of fixed and random effects for the multilevel models, is provided by Anderson [42].

### Software and code

All analysis was conducted within the R Statistical Software environment (version 4.2.2) (https://www.r-project.org/). The *lme4* package [43] was used to fit all models, while model comparisons were conducted via the *pbkrtest* package [44]. Estimated marginal means were calculated using the *emmeans* package [45]. To provide full transparency of the methodology, data and code for one example indicator has been made available *by Soraghan* [46].

### Statement on involvement of lived experience

The presented work constitutes one strand of the CSRR study. Due to deadlines around governmental decision making, the research was subject to tight timeframes and there was not scope to include participatory work with children and families with lived experience of both health and social care services. However, throughout the course of the CSRR study, there was careful consideration of existing evidence which details children, young people and families’ views and experiences of support and services.

## Results

### The association between integration and outcomes

The results from the multilevel model analysis of the association between different forms of HSCP integration and children’s outcome indicators are provided in Table 2, with statistically significant *p*-values highlighted in bold. For 22 of the 25 indicators there was no evidence of an association between the level of structural integration of children’s services and changes in indicator performance of local authority areas after integration.

There was, however, evidence of a statistically significant association for three indicators – namely the conversion rate of case conferences to child protection registrations, the percentage of ‘looked after’ children (i.e. children in care) who had experienced three or more placements in the last twelve months, and the percentage of Primary 1 children aged 4 to 6 who were overweight or obese.

However, further detail on these three statistically significant associations (Table 3) shows that the estimated changes for each indicator were small in scale and/or did not paint a clear picture of the potential impact of integration (i.e. the changes were not directionally consistent across integration categories).

**Table 3:** ‘Average’ (estimated marginal mean) percentage change for significant results in the period after the integration of children’s services.


INDICATOR	NO STRUCTURAL INTEGRATION	PARTIAL STRUCTURAL INTEGRATION	FULL STRUCTURAL INTEGRATION

Case conference to child protection registration conversion rate	+3.3%	+5.8%	+1.9%

Looked after children with 3+ placements in last 12 months	–1.0%	–0.4%	+0.2%

Primary 1 children (4–6 years) who are overweight or obese	–0.2%	+0.7%	–0.6%


This is further highlighted within Figure 2, which depicts the modelled changes for the ‘conversion rate of case conferences to child protection registrations’ indicator across each of the three categories of integration. This indicator was selected as it relates to the levels of multi-agency working and information sharing between practitioners, consequently our theory of change for this indicator was that more integrated structures would be expected to have higher conversion rates. The vertical dashed line represents the year (2016) in which HSCP integration occurred in the majority of local authority areas. The raw data for this indicator (depicted by dashed lines) shows a high degree of variation across different local authority areas both before and after integration. Multilevel models are an ideal tool for dealing with this complexity, as they can account for differing baselines and trajectories across individual areas while simultaneously taking additional contextual factors into consideration.

The modelled trends seen in Figure 2 (depicted by solid lines) illustrate that the changes seen after integration were small within the context of the underlying regional variation, and far smaller than the pre-existing differences between the categories prior to integration, reminding us both that statistical significance does not always equate to practical significance, and that wider factors beyond structural integration (such as differences in local processes, multi-agency working, information sharing and children’s needs, all of which are difficult to measure through administrative data) must be responsible for these pre-existing differences.

Overall, given that there was no statistical evidence of an association in 22 of 25 indicators, and that the observed changes for the remaining three indicators were small in magnitude and not directionally consistent across integration categories, the findings indicate that there is no consistent statistical evidence of an association between the structural integration of children’s services into HSCPs and changes in outcomes for children and young people.

### The influence of other contextual factors

Table 2 additionally provides information on the contextual factors that were found to be associated with changes in each of the outcome indicators. The significance of an association was determined based on the conventional statistical threshold of *p* = 0.05.

In consensus with the existing evidence [33,47,48,49] deprivation was found to be detrimental to children’s outcomes across a host of domains, including health, education and child protection. Changes in 16 of the 25 indicators analysed were significantly associated with the level of deprivation within a local authority area, in what would generally be deemed a detrimental manner. The analysis showed that more deprived areas had, amongst other findings, higher levels of child protection activity; higher absence rates for school pupils; a higher proportion of young people who were not in education, training or employment; and a higher percentage of children aged 27-30 months who had developmental concerns recorded.

Population density was associated with changes in 9 of the 25 indicators. Findings included higher rates of children in temporary accommodation and an increase in the percentage of school leavers going on to positive destinations in more densely populated areas. The effects of population density were more nuanced than those of deprivation, with densely populated areas often seeing more positive outcomes than less populated areas after accounting for the effects of deprivation.

The COVID-19 pandemic and associated public health restrictions were also found to have been influential on children’s outcomes, with changes in 14 of the 25 indicators significantly associated with the pandemic period. In consensus with previous research [37,50], this analysis found evidence of a reduction in statutory activity around child protection (registrations and Children’s Hearings) and a reduction in the number of children entering care. Other changes identified include a reduction in the number of children and young people involved in youth justice processes, an increase in the proportion of young children who had developmental concerns identified by health visitors, and a reduction in the proportion of children registered with a dentist.

There was no relationship found between children’s outcomes and whether local authorities shared a boundary with a health board.

## Discussion

### Can structural integration be discounted as a means of improving children’s outcomes?

The research findings may be seen to challenge the positivist policy assumption that structural integration is the optimal structural arrangement [1,2,3,5]. The study found no evident association between the structural integration of HSCPs in Scotland’s 32 local authority areas and changes in children’s outcome indicators over the time period studied. This finding could be interpreted as structural integration does not matter and should be discounted as a means of improving children’s outcomes. This interpretation would, however, be simplistic.

First, it is important to consider the feasibility of this retrospective approach to understanding the impact of HSCP integration. In the absence of a pre-existing theory of change detailing the changes that we should expect to see from integration and the mechanisms by which this will happen, we cannot be certain that the outcome indicators selected were the most appropriate.

Second, this research has focused on one specific approach to structural integration, i.e. the formation of HSCPs in Scotland. While these structures have not been found to have impacted on children’s outcomes, there may be other forms of structural integration that could. Notwithstanding the short-lived experiment of Children’s Trusts in England [8], what impact would an integrated children’s services structure spanning child health, social care, social work, early learning and childcare, and education have on children’s outcomes? It is possible that structural integration could have a role to play in improving children’s outcomes, provided the ‘right’ services are integrated into the ‘right’ structure and are accompanied by the ‘right’ conditions that support effective multi-agency working. Indeed, the inclusion of children’s social care and social work services was optional for Scotland’s HSCPs and, perhaps relatedly, a criticism that could be levelled at them is that children’s needs have been peripheral in the face of increasing adult health and social care needs. Given this, it is perhaps not surprising that this discretionary form of structural integration has not directly impacted on children’s outcomes.

Third, while the research has sought to assess impact at least 5 years after the establishment of HSCPs, large-scale structural reforms take time to be implemented, with a 10-year timeframe more widely referred to [26]. It takes many years for changes to structures to translate into how those interacting with the services experience them, which will in turn take more time to translate to changes in outcomes. The limited time elapsed since HSCP integration means this analysis cannot provide a definitive assessment of the impact of HSCPs on children’s outcomes. The research does, however, offer a statistical methodology that can be used to help assess impact in the years to come.

### The challenge of attribution within a complex environment

The multilevel modelling did not find consistent evidence of a relationship between changes in the outcome indicators and structural integration. Associations were, however, found with wider economic, social and health factors, specifically deprivation, population density and the COVID-19 pandemic. This raises the question of whether change can be attributed to a single structural reform or intervention within a complex system characterised by a myriad of unpredictable internal and external factors. Cook [6] argues that direct *attribution* is not possible within a complex system, and we should be looking to understand the *contribution* of a given reform instead. Multilevel modelling provides an appropriate framework through which the contribution of different factors can be considered. Irrespective of the challenge of attribution or contribution, structural reforms should not be viewed as an all-encompassing solution to improving outcomes for children and young people. Other policy objectives would appear to be more important, not least tackling area deprivation and child and family poverty. The longer-term impacts of the COVID-19 pandemic must also continue to be monitored and responded to.

### The importance of a theory of change to understand and assess impact

Theories of change provide clarity on the causal chain, detailing how the change being implemented will lead to the desired outcome. Articulating a theory of change is particularly important when designing and implementing a reform within a complex system as it supports greater focus on the key levers of change. However, despite their value, the research team was unable to source a theory of change for HSCPs in Scotland. The closest approximation was reference to nine adult outcome indicators that HSCPs were expected to impact on [17,30]. The lack of a guiding theory of change is not specific to Scotland as McTier [26] found that theories of change were also lacking for the transformational change programmes studied in Finland, the Netherlands, New Zealand, Northern Ireland, and the Republic of Ireland.

In the absence of a theory of change that sets out the process, output and outcome indicators that would be impacted by HSCPs, the research team had to conceive of its own. The 25 children’s outcomes indicators used were therefore (in part) selected because the research team hypothesised that the indicators could be impacted by structural integration. This makes the statistical modelling described in this article a retrospective ‘best fit’ activity rather than an exercise that has been intentionally planned from the outset of HSCP integration efforts in order to assess their impact. The absence of a theory of change means it is possible that key objectives of HSCP integration may not have been understood by the research team and not then factored into the statistical indicators selected.

### The need to address gaps in the administrative data collected

The statistical modelling used in this article was dependent on the administrative data collected. The use of 25 children’s outcomes indicators was a strength of the research, particularly as it demonstrated how the administrative data routinely collected by Scotland’s children’s services can be applied in a different setting. However, there were gaps in the available data that would have enhanced the research. These included gaps in data relating to early help and prevention for children at risk (such as referrals of children to social work services), the health of older children and young people, the extent to which children feel safe, cared for and included, and the wellbeing of the children’s services workforce. There was also limited data disaggregated to different groups of children and young people (e.g. disabled children) to understand how structural integration might be impacting on key beneficiary groups. These gaps in the available evidence do not appear to be unique to Scotland, with many of these areas also identified by the Department of Education in England as areas that require further data development [51].

### The need for different types of data

Many of this study’s children’s outcome indicators are arguably better categorised as *process* indicators that tell us more about the way in which services are operating as opposed to the children’s actual outcomes, wellbeing and experiences of services. In light of this, administrative data alone may be insufficient to assess the impact of structural integration. While more challenging to collect, experiential data from children would provide additional insight into why and how they were engaging with services, and how the interaction and/or support provided by services was experienced by them. The policy assumption would be that more integrated structures would enable different services to work more closely around individual children and consequently their experience of the support received would be more positive.

Given what is known about the time needed for structural integration to translate into changes in service delivery, and consequently into changes in the experiences and outcomes of those engaging with the service, the collection of experiential data should also extend to the workforce. Aligned to a theory of change, data collection should seek to capture the changes to practice and service delivery that have stemmed from structural integration (for example, shared language and culture across integrated services, or the extent to which robust processes for information sharing are in place), rather than seek to immediately assess changes in children’s experiences and outcomes. Workforce experiential data can be seen as a precursor to determining whether outcomes are likely to change by providing evidence of whether integration is being implemented as intended, as well as providing insights that can drive improvement.

### The importance of considering other forms of integrated working

This research has focused on a particular form of integration but integration takes many different forms [1,2,52,53] and it may be that other forms of integrated working have a more evident impact on children’s outcomes than changes to structural arrangements. For example, effective multi-agency integration at operational management or frontline practitioner levels, including the co-location of services, multi-disciplinary teams, information sharing or pooling of budgets, may enable different services to work better together to respond to the needs of individual children.

Defining, categorising and measuring other forms of integrated working is a much more complex task than this research’s categorisation of structural integration across Scotland’s HSCPs. Integrated working may be most strongly experienced at the community level in the form of local multi-agency teams, hubs or clusters, however few routine children’s outcome indicators are available at the sub-local authority area level. Indeed, for this study, several outcome indicators were unable to be included as they could not be disaggregated from regional health board level to local authority level, let alone community level.

### Limitations

In addition to the retrospective nature of the study and the lack of an HSCP theory of change, there are specific limitations to the statistical analysis which should be considered when interpreting the findings. First, it is likely that a five-year follow-up period is insufficient for the evaluation of a large-scale reform such as the creation of HSCPs. Second, Scotland’s 32 local authority areas represent a comparatively small sample size for modelling purposes, thereby limiting the power of the statistical models to detect significant relationships. Third, the gaps in the available data (e.g. early help and workforce data) mean that there may be areas of change that were not captured within this research. In addition, administrative data is collected for operational as opposed to research purposes, and as such is prone to errors and inconsistencies in collection, particularly across differing localities and time periods. As such, while we did not find evidence of a relationship between structural integration and outcomes, we cannot definitively state that such a relationship does not exist.

## Conclusions

While assessing the impact of integration is a complex task, it is important that long-standing assumptions regarding the benefits of increased levels of service integration are empirically tested. The statistical methods presented here provide a framework within which changes to outcomes can be assessed to evaluate the efficacy of a reform. The approach can account for the influence of wider environmental factors and is flexible to situations where there has been variation in the implementation of a reform across different geographical regions.

However, the article has also highlighted the challenges of retrospectively assessing the impact of complex systems-level change using routinely collected administrative data only. Other information sources are needed to provide more thorough assessment of structural integration impacts upon children’s outcomes, not least an underlying theory of change and experiential data from children, young people and the children’s services workforce.

## Data Accessibility Statement

Data for many of the outcome indicators was publicly available and the data sources for these are provided in Appendix A within the supplementary files. Seven of the indicators were bespoke and provided directly to the research team for the purposes of this study by the Scottish Government, Public Health Scotland and Scottish Children’s Reporter Administration. As such, the data for those seven indicators cannot be shared. Data and code to replicate the analysis for one example indicator is provided [46].

## Additional Files

The additional files for this article can be found as follows:

10.5334/ijic.9145.s1 Supplementary File 1. Appendix A.

10.5334/ijic.9145.s2 Supplementary File 2. CSSR Strand 3 Example Code and Data.
