# Supplementary material for: The Impact of Health and Social Care Integration on Children and Young People’s Outcomes: What Can Be Determined from Scotland’s Administrative Data?
Source: Int J Integr Care. 2025 Nov 26;25(4):17. doi: 10.5334/ijic.9145 (PMC12662158; doi:10.5334/ijic.9145)
Supplement: Supplementary File 1. — Appendix A. [file ijic-25-4-9145-s1.pdf]

The impact of health and social care integration on children and young people's outcomes: What can be determined from Scotland's administrative data?

Appendix A

Table A.1: Data sources for each of the outcome indicators for children and young people

**CHILD PROTECTION**	**Data owner†**	**Data source***
Child protection registrations (including pre-birth)	SG	https://www.gov.scot/collections/childrens-social-work/
Child protection de-registrations	SG	https://www.gov.scot/collections/childrens-social-work/
Case conferences to child protection registration conversion rate (0-15 years)	SG	Indicator provided to the research team by the data owner
Hearings arranged for children for non-offence grounds per 10,000	SCRA	Indicator provided to the research team by the data owner
**YOUTH JUSTICE**		
Children referred to Children's Reporter on offence grounds	SCRA	https://www.scra.gov.uk/stats/
Children and young people aged 12 to 20 proceeded against	SG	https://www.gov.scot/publications/criminal-proceedings-scotland-2020-21/documents/
**'LOOKED AFTER' CHILDREN**		
Children starting to become looked after	SG	https://www.gov.scot/collections/childrens-social-work/
Children starting to be looked after at home as proportion of all children	SG	https://www.gov.scot/collections/childrens-social-work/

becoming looked after		
Children ceasing to be looked after (0-15 years)	SG	Indicator provided to the research team by the data owner
Children aged 0-15 leaving care to return home	SG	Indicator provided to the research team by the data owner
Children with 3+ placements in last 12 months	SG	Indicator provided to the research team by the data owner
Looked after school leavers with 1+ qualifications as SCQF level 4	SG	https://www.gov.scot/publications/education-outcomes-looked-children-2020-21/
Looked after school leavers with a positive follow-up destination	SG	https://www.gov.scot/publications/education-outcomes-looked-children-2020-21/
School attendance for looked after children	SG	Indicator provided to the research team by the data owner
**EDUCATION AND EMPLOYABILITY**		
Unauthorised absence rates of primary school pupils	SG	https://www.gov.scot/publications/school-attendance-and-absence-statistics/
Unauthorised absence rates of secondary school pupils	SG	https://www.gov.scot/publications/school-attendance-and-absence-statistics/
16-19 year olds not in education, training or employment	SDS	https://www.skillsdevelopmentscotland.co.uk/publications-statistics/statistics/annual-participation-measure
**HEALTH**		
Teenage pregnancy rate per 10,000 aged 0-17	PHS	https://publichealthscotland.scot/publications/teenage-

		pregnancies/teenage-pregnancies-year-of-conception-ending-31-december-2020/
Primary 1 children (4-6 years) overweight or obese	PHS	https://www.opendata.nhs.scot/dataset/primary-1-body-mass-index-bmi-statistics
Children (0-17 years) registered with an NHS dentist	PHS	Indicator provided to the research team by the data owner
27-30 month old children reviewed by health visitors	PHS	https://www.opendata.nhs.scot/dataset/27-30-month-review-statistics
27-30 month old children with a developmental concern	PHS	https://www.opendata.nhs.scot/dataset/27-30-month-review-statistics
**HOUSING**		
Children associated with applications assessed as homeless or threatened with homelessness	SG	https://www.gov.scot/publications/homelessness-scotland-2020-2021/documents/
Children in temporary accommodation	SG	https://www.gov.scot/publications/homelessness-scotland-2020-2021/documents/
**WORKFORCE**		
Whole-time equivalent rates for social workers in fieldwork services for children per 100,000	SSSC	https://data.sssc.uk.com/local-level-data/162-interactive-social-worker-data-tool

†SG - Scottish Government, SCRA - Scottish Children's Reporter Administration, SDS - Skills Development Scotland, PHS – Public Health Scotland, SSSC – Scottish Social Services Council

*All links accessed on 17th February 2025.
